# Non-Alcoholic Fatty Liver Disease in HIV/HBV Patients – a Metabolic Imbalance Aggravated by Antiretroviral Therapy and Perpetuated by the Hepatokine/Adipokine Axis Breakdown

**DOI:** 10.3389/fendo.2022.814209

**Published:** 2022-03-09

**Authors:** Simona Alexandra Iacob, Diana Gabriela Iacob

**Affiliations:** ^1^ Department of Infectious Diseases, Carol Davila University of Medicine and Pharmacy, Bucharest, Romania; ^2^ Department of Infectious Diseases, National Institute of Infectious Diseases “Prof. Dr. Matei Bals”, Bucharest, Romania; ^3^ Department of Infectious Diseases, Emergency University Hospital, Bucharest, Romania

**Keywords:** non-alcoholic fatty liver disease, hepatitis B virus, HIV, hepatokines, adipokines, oxidative stress, metabolic syndrome, antiretroviral treatment

## Abstract

Non-alcoholic fatty liver disease (NAFLD) is strongly associated with the metabolic syndrome and is one of the most prevalent comorbidities in HIV and HBV infected patients. HIV plays an early and direct role in the development of metabolic syndrome by disrupting the mechanism of adipogenesis and synthesis of adipokines. Adipokines, molecules that regulate the lipid metabolism, also contribute to the progression of NAFLD either directly or *via* hepatic organokines (hepatokines). Most hepatokines play a direct role in lipid homeostasis and liver inflammation but their role in the evolution of NAFLD is not well defined. The role of HBV in the pathogenesis of NAFLD is controversial. HBV has been previously associated with a decreased level of triglycerides and with a protective role against the development of steatosis and metabolic syndrome. At the same time HBV displays a high fibrogenetic and oncogenetic potential. In the HIV/HBV co-infection, the metabolic changes are initiated by mitochondrial dysfunction as well as by the fatty overload of the liver, two interconnected mechanisms. The evolution of NAFLD is further perpetuated by the inflammatory response to these viral agents and by the variable toxicity of the antiretroviral therapy. The current article discusses the pathogenic changes and the contribution of the hepatokine/adipokine axis in the development of NAFLD as well as the implications of HIV and HBV infection in the breakdown of the hepatokine/adipokine axis and NAFLD progression.

## 1 Introduction

Non-alcoholic fatty liver disease (NAFLD) encompasses a spectrum of pathological changes induced by the accumulation of fat in the liver parenchyma, in the absence of alcohol consumption. According to the literature, NAFLD is encountered in 25% of the general population and represents the most common liver-related disease, especially in developed countries (1, 2). From a histopathological perspective NAFLD progresses from the simple accumulation of fat (fatty liver or steatosis) to liver inflammation (non-alcoholic steatohepatitis, NASH) and to liver fibrosis, potentially leading to hepatocellular carcinoma (HCC) (3). The deposition of fat in the liver is a reversible process, while NASH is an aggressive form of NAFLD leading to cirrhotic transformation and carcinogenesis.

As much as 27-44% of patients with NAFLD display NASH on liver biopsy and 30-42% of these have been shown to progress to liver fibrosis depending on various factors such as age, gender, geographical location or other comorbidities (4–7). However, the high prevalence of NAFLD and its close relation with the metabolic syndrome (MetS) (8) increase the risk of mortality and morbidity during NAFLD (8). The outcome of NAFLD is further aggravated by HIV and HBV infections, as a result of the intrahepatic inflammatory response and metabolic imbalances triggered by both viruses (9, 10). The pathological changes behind these events consist in the ability of viruses to simultaneously control key enzymes needed for viral replication and transcription factors involved in metabolic processes.

The HIV/HBV viral replication, as well as the inflammatory response and fatty deposition within the liver contribute to hepatic mitochondrial toxicity, one of the main mechanisms responsible for the development of NAFLD. Additionally, the antiretroviral (ARV) drugs induces a spectrum of metabolic abnormalities strongly associated with NAFLD known as HIV/ART–associated lipodystrophy syndrome (HALS). The limited ability of the liver to coordinate all these events through the hepatokine/adipokine network enables the progression of liver lesions and aggravates the ensuing metabolic imbalance.

Data related to the evolution of the hepatokine/adipokine axis in HIV and HBV infected patients with NAFLD are scarce. Currently no review has previously approached the mechanisms through which the hepatokine/adipokine axis controls the liver impairment induced by HIV/HBV. The article sums up available data on the immune and metabolic implications of the hepatokine/adipokine axis in HIV/HBV-infected patients with NAFLD. On the long term, the modulation of hepatokine/adipokine axis represents an important direction for research and could play a significant therapeutic benefit towards the attenuation or prevention of NAFLD

The article is structured in two parts. The first part discusses the cellular mechanisms and the contribution of the hepatokine/adipokine axis in the development of NAFLD, whereas the second part presents the implications of HIV/HBV infections on these mechanisms.

## 2 Cellular Mechanisms Involved in the Development of NAFLD and the Role of the Oxidative Stress

The liver holds a central role in metabolic homeostasis given its key function in the fat and glucose metabolism, namely in fat absorption and fatty acid (FA) metabolism, in the conversion of glucose to glycogen and vice versa and in the regulation of insulin signals from liver receptors. Hence, the resulting liver-related lesions during NAFLD are closely linked with additional metabolic changes and particularly with the progression of the MetS. NAFLD could favor the development of type 2 diabetes (T2D), insulin resistance (IR), atherogenic dyslipidemia and obesity, all inducing MetS (11) while at the same time, these conditions are risk factors for the evolution of NAFLD (12, 13). In this respect, both hyperinsulinemia and the excessive accumulation of triglycerides (TGs) in the adipose tissue interfere with liver lipogenesis and *de novo* lipogenesis (DNL) and contribute to the onset of steatosis, the first stage of NAFLD (14). Fatty deposition within the liver cells gradually induces mitochondrial oxidative damage and generates oxidative stress (OxS), a fundamental cellular process in the development of NAFLD (15).

OxS represents a cellular imbalance between free radicals (reactive oxygen species, ROS) and antioxidants accompanied by the reduction of the oxidative capacity and antioxidant response in the mitochondria, and subsequently in the endoplasmic reticulum. The OxS response involves the activation of numerous transcription factors which upregulate the inflammatory response and modulate the glycolipid metabolism, further favoring the occurrence of NASH (16). In moderate amounts, ROS play a key role as signalling molecules that control the immune response and protect against invasive pathogens (17). However, the overproduction of ROS in the liver parenchyma generates an inflammatory response that triggers cell necrosis and subsequently favors liver fibrosis (18). ROS also initiate a process of oxidative damage of the polyunsaturated FA belonging to the cell membranes through lipid peroxidation and further release toxic intermediate products, namely the lipid hydroperoxides. The accumulation and continuous conversion of lipid hydroperoxides to alkoxyl and peroxyl radicals aggravates all the more the oxidative damage of cells and membranes and facilitates the release of ROS and mitochondrial toxicity in a vicious circle (19). The ensuing mitochondrial toxicity further contributes to liver inflammation and fibrosis (20, 21). In addition, hydroperoxides diffuse across cell membranes and serve as pancreatic signalling molecules to influence glucose-stimulated insulin secretion and to suspend the pancreatic glycemic control (22). All these aspects highlight the close link between OxS, liver inflammation and glycolipid metabolism.

## 3 Hepatokine/Adipokine Axis in NAFLD Pathogenesis

### 3.1 Overview

Currently, there is ample evidence that NAFLD is a multifactorial disorder dependent on metabolic, genetic, environmental, toxic and infectious factors in different combinations (“multiple-hits” theory) (23). NAFLD develops as a stress response to these factors. Recently, the pathogenesis of NAFLD has been associated with the release of “organokines”, peptides that are synthesized within the liver or in various tissues. Notably, these organokines play an active role in the regulation of metabolic and inflammatory processes and connect numerous tissues/organs, particularly the liver and the adipose tissue.

Organkines are secreted in various physiological or pathological conditions predominantly in the liver (“hepatokines”), adipose tissue (“adipokines”) or muscles (“myokines”).Various authors have described the functions and features of these structures, their connections and their involvement in NAFLD (24–27). Some of these bioactive peptides are currently studied as therapeutic targets or biomarkers of NAFLD severity (28). However, the number and specific functions of these regulatory peptides have not been elucidated and their role in the pathogenesis of NAFLD remains unclear. Currently, the adipose tissue is considered the largest endocrine organ, producing over 700 adipokines of which only a few have been characterized. Two of these adipokines, namely adiponectin and leptin have been validated by clinical and histological studies in NAFLD (26, 29). Additionally, hepatocytes secrete more than 500 proteins of which only a small number have been studied and very few proteins such as fetuins A or FGF21 have been clearly correlated with distinct manifestations of MetS or NAFLD (30).

### 3.2 Cellular Mechanisms Activated by Hepatokines and Adipokines

Hepatokines and adipokines carry multiple cellular functions, regulating various transcriptional factors, receptors or key enzymes connected to metabolic, immune, antiviral, or antitumor processes. Hence, these organokines control the transcription factors that interfere in the regulation of insulin or lipid signalling (31–33) (e.g. carbohydrate response element binding protein- ChREBP or sterol regulatory element-binding transcription factor 1-SREBP-1c), as well as in hepatic inflammatory response, fibrogenesis or carcinogenesis (34–37) (e.g., nuclear factor kappa-light-chain-enhancer of activated B cells-NF-kB or members of the signal transducer and activator of transcription*-*STAT family). Adipokines and hepatokines can also activate specific receptors (e.g AdipoR1/R2, ChemR, LepRb) (38–41) or metabolic receptors (e.g. the peroxisome proliferator-activated receptors-PPAR family) (33, 42–47). Additionally, certain organokines (adiponectin, resisitin, fetuin A) could display a competitive binding to the cellular receptors TLR4/CD14, which transduce the bacterial lipopolysaccharide (LPS) signal into inflammatory signals (48–51). Some organokines such as adiponectin and fibroblast growth factor 21 (FGF21) impair the metabolic activity of c-Jun N-terminal kinase*s* (JNK). Also leptin, ghrelin, adiponectin or FGF21 dysregulate the signalling pathways of mammalian target of rapamycin (mTOR1), a protein kinase that coordinates lipid homeostasis and cellular growth but also, liver inflammation and carcinogenesis (52). Furthermore, the contribution of organokines in liver pathology is highly intricate as a result of the numerous synergistic or antagonistic interactions that are established between them. The main hepatokines and adipokines which contribute to NAFLD and their mechanism of action is presented in the **Table 1**. The interactions of these organokines are presented in the **Table 2**.

**Table 1:** Cellular targets regulated by adipokines and hepatokines with relevance in the pathogenesis of non-alcoholic fatty liver disease and metabolic syndrome.

*Cellular targets controlled by hepatokines and adipokines*	*Action mechanisms involved in the pathogenesis of NAFLD*	*Hepatokines and adipokines that control the cellular targets*
***1 .TRANSCRIPTION FACTORS***		
*a) Nuclear factor kappa-light-chain-enhancer of activated B cells (NF-kB)*	The excessive release of NF-kB promotes NAFLD through multiple mechanisms (53): a) the activation of liver fibrosis in HSCs; b) the initiation of the inflammatory response in KCs; c) the releasing of inflammatory key cytokines (TNF-α, IL6); d) anti-apoptotic functions and the involvement in hepatocarcinogenesis; e) the inflammatory response during LPS stimulation; f) the promotion of IR (54)	***FGF21, ghrelin, resistin, fetuin A ghrelin*** (34–37)
*b) Signal transducer and activator of transcription (STAT)*	Members of the STAT protein family modulate liver inflammation and fibrosis (55, 56) and also play a defining role in the antiviral and antitumoral immune response (57)	***Leptin, adiponectin and FGF21*** (38, 58–60)
*c) Carbohydrate response element binding protein (ChREBP) Sterol regulatory element binding protein (SREBP-1c)*.	ChREBP and SREBP-1c play a synergic role and regulate the genes expression of glycolytic and lipogenic pathways (61); ChREBP is stimulated by glucose; SREBP-1c is activated by insulin. Both factors regulate FA.synthesis. The upregulation of these factors favors hepatic steatosis, IR and the progression of MetS (62, 63)	***FGF21, adiponectin and leptin*** (31–33)
***2. RECEPTORS***		
***A) Receptors activated by various ligands, including organokines***		
*a) Peroxisome proliferator activated receptors (PPARs).*	The activation of PPARs attenuates the development of NAFLD through its regulation of the lipid metabolism and reducing IR (PPAR-*α*, PPAR-*γ)* or through the attenuation of liver inflammation (PPAR-β/δ*)* (64)	***FGF21, leptin, adiponectin and ghrelin)*** (33, 42–47)
*b) The complex of toll like receptor 4 (TLR4) and CD14 receptor*	TLR4 is a key receptor of KCs and adipose tissue involved in the activation of the inflammatory response.TLR4 signalling is amplified by OxS and coupled with lipid metabolism (50); CD14 is a co-receptor of TLR4 which facilitates the binding of LPS and the release of cytokines that are dependent of NF-kB (65). TLR4/CD14 signalling favors the progression of NAFLD through NF-kB activation, the release of proinflammatory cytokines (TNF-a,IL6) (65, 66), IR (67, 68) and triglycerides accumulation (69)	***Resistin, fetuin A adiponectin. Fetuin A is a ligand for TLR4, also binding to FA*** (48–51)
***B) Specific receptors***	Leptin, chemerin, adiponectin and ghrelin receptors	***Leptin, chemerin, adiponectin and ghrelin*** (38–41)
***3. KEY ENZYMES***		
a) *Mammalian target of rapamycin complex 1 (mTORC1)*	mTORC1 promotes SREBP-dependent lipogenesis (70) and modulates the immune response under cellular stress. The dysregulation of mTORC1 favors liver steatosis (71) through the activation of SREBP-dependent lipogenesis and also hepatic carcinogenesis due to the worsening of OxS and inflammatory response (71)	***Ghrelin, FGF21, adiponectin and leptin*** (39, 72–74)
b) *c-Jun N-terminal kinases (JNKs)*	JNK promotes the development of NAFLD through favorable effect towards hepatic steatosis, inflammation, fibrosis, IR and obesity (75)	***Adiponectin leptin and FGF21*** (38, 74, 76, 77)

NAFLD, nonalcoholic chronic liver diseases; IR, insulin resistance; HSCs, hepatic stellate cells; KCs, Kupffer cells; LPS, lipopolysaharide; FA, free fatty acids; MetS, metabolic syndrome; OxS, oxidative stress.

**Table 2:** Correlations between various hepatokines and adipokines with a possible role in the pathogenesis of non-alcoholic fatty liver disease.

***Fetuin A/adiponectin axis*** *-antagonistic relationships*	Fetuin A represses adiponectin and the vice versa, adiponectin inhibits hepatic fetuin A expression *via* the AMPK-NFκB pathway (78)
***Adiponectin/leptin axis*** *-antagonistic relationships*	Adiponectin inhibits the synthesis of leptin in liver carcinoma (79)
***Adiponetin/FGF21 axis*** *- reciprocal stimulation*	Adiponectin stimulates FGF21 while FGF21 increases the expression of adiponectin; some of the FGF21 effects are thought to be mediated by adiponectin (80, 81). However, in the adipose tissue, FGF21 suppresses adiponectin and promotes leptin (82).
***Leptin/FGF21 axis*** *-reciprocal stimulation*	Leptin increases FGF21 secretion (83) while FGF21 mediates the effects of leptin; both organokines are modulated by the same transcriptional factors namely PPARα and PPARγ (45, 46). Also various roles of leptin actions are probably due to the FGF21 and leptin resistance could actually be mediated by the resistance to FGF21 (83).
***Selenoprotein P/adiponectin axis*** *-antagonistic relationships*	Negative correlations of selenoprotein P with adiponectin in type 2 diabetes patients (84)
***Resistin/adiponectin axis–*** *antagonistic relationships*	Resistin inhibits adiponectin; possible role in the pathogenesis of NAFLD (85)
***Chemerin**- reciprocal stimulation with FGF21* (86) *and adiponectin* (87) *and leptin* (88)	Chemerin might be the link between obesity and NAFLD (88)

### 3.3 NAFLD Pathogenic Pathways Regulated by Hepatokines and Adipokines

**Figure 1:**
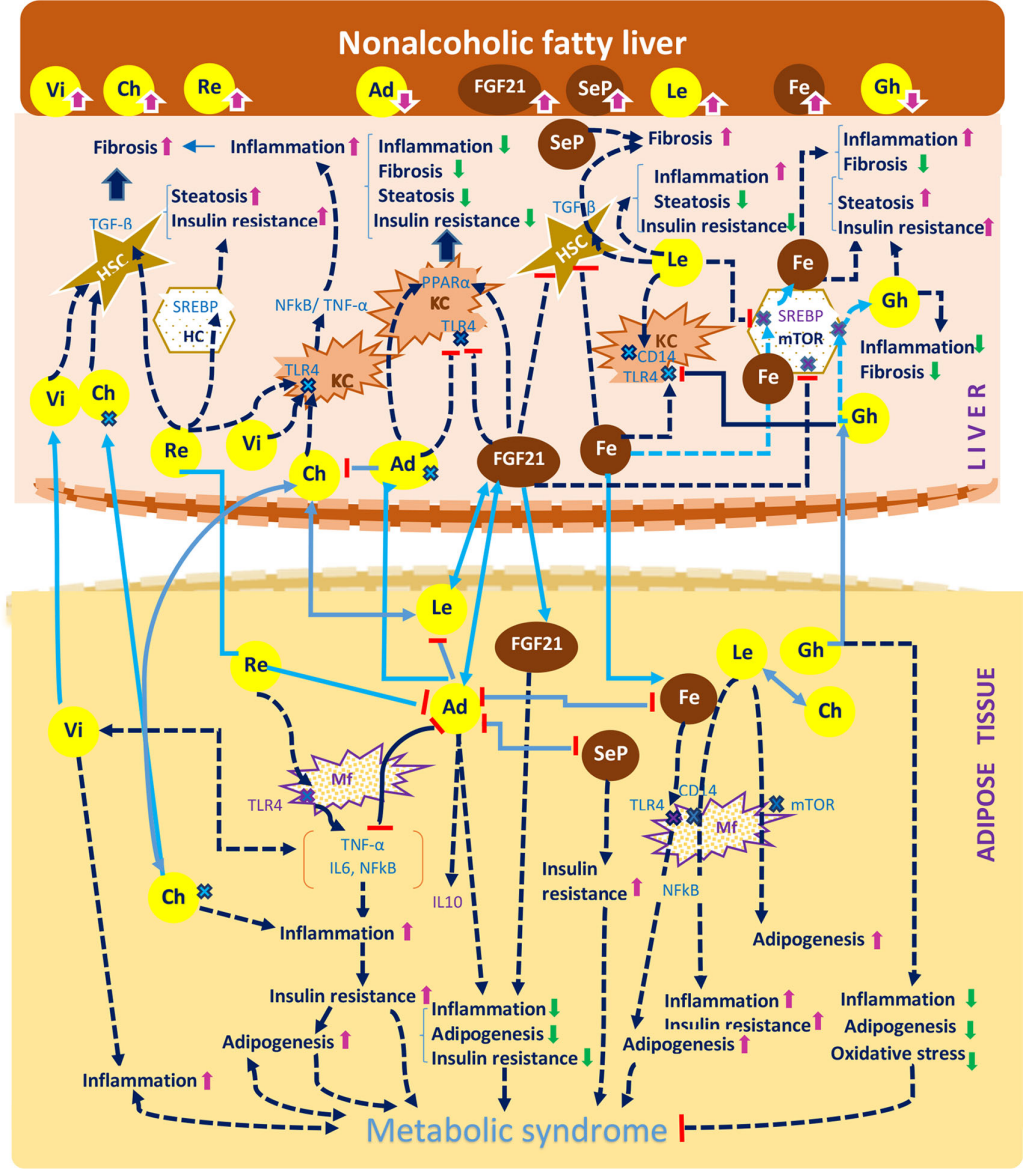
A concise representation of the hepatokines and adipokines presented in the article and their implications in the pathogenesis of non-alcoholic fatty liver disease according to the current studies. The diagram indicates the effect of hepatokines (FGF21, selenoprotein P, fetuin A, chemerin) and of the adipokines (visfatin, resistin, adiponectin, ghrelin), against the mechanisms that drive the pathogenesis of NAFLD, namely steatosis, inflammation, fibrosis and insulin resistance and also against the development of the metabolic syndrome, namely adipogenesis, inflammation and insulin resistance. The diagram depicts the following processes: a. The effects of organokines on liver inflammation: visfatin, chemerin, leptin, resistin, fetuin A activate the NF-kB and TNF-α/IL6 pathway and induce a proinflammatory effect mediated by Kuppfer cells (KCs). The previous organokines exhibit high concentrations in NAFLD. By comparison, adiponectin, FGF21 and ghrelin exert an anti-inflammatory effect. b. The effects of organokines on liver fibrosis: visfatin, chemerin, leptin, selenoprotein P mediate the release of TGF-β in hepatic stelatte cells (HSCs), while adiponectin and FGF21 exert an antifibrotic effect. c. The additive effect of organokines against the evolution of the metabolic syndrome: visfatin, chemerin and leptin promote the metabolic syndrome through their proinflammatory and proadipogenic effect, as well as through their role in the aggravation of insulin resistance. On the other hand, adiponectin and FGF21 play a protective role against the metabolic syndrome. Organokines can stimulate each other (e.g: adiponectin and leptin with FGF21) or inhibit each other (e.g: fetuin A, leptin, selenoprotein P, resistin with adiponectin). Organokines synthesized predominantly in the liver are presented in brown and those synthesized predominantly in the adipose tissue are presented in yellow. The correlations between these different organokines are shown in blue. The serum concentrations of these organokines in NAFLD (high or low) are represented by arrows. Vi, visfatin; Re, resistin; Fe, fetuin A; Le, leptin; Ch, chemerin; Ad, adiponectin; Gh, ghrelin; SeP, selenoprotein; Re, resistin; Mf, macrophage; HC, hepatic cell; 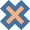 receptor.

Hepatokines and adipokines act as intercellular signals which modulate the liver lipogenic, inflammatory and fibrogenic pathways (25, 89). Lipogenic pathways are modulated by hepatokines and adipokines through their specific control over the FA flux, DNL and insulin signals. These carbohydrate-dependent processes are regulated through the activation of PPAR-α/γ receptors (64) as well as by transcription factors and enzymes involved in the lipid homeostasis (e.g. mTOR/SREBP1c and ChREBP) (25, 90). A predominant lipogenic effect can be observed in the case of resistin, ghrelin, fetuin A, chemerin and selenoprotein P (SeP). Inflammatory and fibrogenic pathways are modulated mainly through activation/inhibition of proinflammatory transcription factors (e.g NF-κB expression or STAT-3), thus modifying the profile of the released cytokines (53, 57, 75). Leptin and resistin are significantly associated with liver inflammation and leptin, resistin, chemerin, fetuin A and SeP with a profibrotic effect. The main adipokines and hepatokines presented in this article along with their metabolic, inflammatory and fibrogenic pathways are depicted in **Table 3** and their potential effect in NAFLD are represented in **Table 4** and **Figure 1**. As shown in **Figure 1**, visfatin chemerin, resisitin and SeP aggravate NAFLD through multiple pathways including the activation of receptors (TLR4/CD14), the regulation of transcription factors ((NF-κB) and the release of inflammatory or profibrotic cytokines (TNF*-α*/IL-6, respectively TGF-β) (98, 99, 104, 123, 124, 126, 132, 150, 154–156). These pathways involved in the control of liver inflammation are mediated by Kupffer cells (KCs) and hepatic stellate cells, (HSCs) and are associated with the exacerbation of inflammation, adipogenesis and finally, with the development of MetS (24, 99, 150, 157–161). By comparison, adiponectin and FGF21 display a hepatoprotective activity (34, 76, 111, 162–164) and attenuate the MetS (24). Various organokines play dual roles. For example, leptin reverses steatosis through SREBP/mTOR inhibition, while also promoting liver inflammation and fibrosis in non-parenchymal cells (79, 96, 165). Additionally, leptin promotes adipogenesis and the immune response and favors MetS (95) Other examples include ghrelin (39, 108) and fetuin A (51, 118, 166) which concurrently aggravate hepatic steatosis and prevent liver fibrosis. Similarly, most adipokines and hepatokines display contradictory roles regarding the progression of NAFLD (27, 167) (**Tables 3** and **4**). In this regard, resistin, a pro-inflammatory adipokine acting *via* NF-κB/TLR4 mediated pathway and overexpressed in NASH (98) can still promote an anti-inflammatory response in the presence of LPS (100, 137, 168). Fetuin A, a hepatokine which induces MetS has been associated with a controversial role in liver fibrosis (119, 120) similar to chemerin in liver inflammation (86, 124). The level of visfatin, a proinflammatory and anti-steatosis adipokine (148) has been correlated with exacerbation as well as protection of liver inflammation (150) while it lacked a correlation with NAFLD in certain studies (146, 151). SeP, a hepatokine shown to display a protective antioxidant role in all stages of NAFLD, with a therapeutic benefit (132), has nevertheless been incriminated in liver fibrosis and IR (129). All of these conflicting aspects underline the need to clarify the roles of hepatokines and adipokines in the evolution of NAFLD and in particular, the need for studies with comparable designs and entry criteria regarding the patient’s metabolic status, age and NAFLD staging (29).

**Table 3:** The main hepatokines and adipokines and their mechanisms in the development of non-alcoholic fatty liver disease.

*Organokine (main source and target tissue)*	*Liver lipogenesis and metabolic syndrome (effect, mechanism)*	*Liver inflammation (effect, mechanism)*	*Liver fibrosis (effects, mechanism)*
***Adiponectin*** *(adipose tissue, liver)* (41, 76, 91–94)	Liver lipogenesis; DNL; FA β-oxidation; PPAR-α expression; Gluconeogenesis; Insulin resistance; Oxidative stress; SIRT-1 activity; SREBP-1 expression;	Liver inflammation; TNF-α/IL6 expression; NF-κB expression; IL-10 expression; AMPK activity;	Liver fibrosis; HSCs activity; TGF-β expression; JNK inhibition
***Leptin*** *(adipose tissue, distributed and active in various tissues including liver)* (41, 93, 95–97)	Liver lipogenesis; FA β-oxidation; PPAR-α activation; Insulin resistance; mTOR activity; SREBP-1/ChREB expression; Adipogenesis;	Liver inflammation; CD14 expression on KCs; STAT-3 activation;	Liver fibrosis; HSCs activity; TGF-β expression; JAK-STAT pathway; HCC risk;
***Resistin*** *(adipose tissue, inflammatory cells including KCs, HSCs; hepatocytes)* (92 *, * 98–107)	Liver lipogenesis; Adipogenesis; Insulin resistance; SREPB-1/ChREBP expression; MetS risk	Liver inflammation; TNF-α/IL6 expression; TLR4/NF-kB-mediated pathway	Liver fibrosis; HSCs activity; TGF-β synthesis; NF-kB signalling;
***Ghrelin*** *(entero-endocrine cells, stomach, pancreas)* (39, 92, 108–110)	Liver lipogenesis; TG serum level; Adipogenesis; T2D risk; Insulin resistance;	Liver inflammation; NF-κB activation; TLR4 expression; IL-10 synthesis; mTOR/PPARγ signalling;	Liver fibrosis; TGF-β synthesis; NF-κB signalling;
***FGF 21*** *(liver/effect on adipose tissue, less other tissues)* (111–113 *, * 114–117)	Liver lipogenesis; FA β-oxidation; mTOR activity; PPAR-α/γ.> Insulin resistance; Adipolysis< Dyslipidemia;	Liver inflammation; NF-κB activation;	Liver fibrosis; TGF-β synthesis;
***Fetuin A*** *(hepatocytes)* (51, 118–122)	Liver lipogenesis; Adipogenesis; SREBP1c; Insulin resistance; T2D risk; Dyslipidemia;	Liver inflammation*; Endogenous ligand between FA and TLR4 TLR4 signaling; NF-κB activation;	Liver fibrosis*; TGF-β activity;
***Chemerin*** *(adipose tissue, liver; receptors in various tissues)* (102, 123–125)	Liver lipogenesis; Adipogenesis; Insulin resistance;	Liver inflammation*; IL-6 expression;	Liver fibrosis; TGFβ1 synthesis;
***Visfatin*** *(adipocytes, hepatocytes, muscle cells, leukocytes)* (92, 126–128)	Liver lipogenesis*; Adipogenesis; Insulin resistance; *	Liver inflammation*; TNF-α/IL-6 synthesis; STAT3/NF-*κ*B pathways;	Liver fibrosis*;
***Selenoprotein P*** *(cellular enzymes with antioxidant properties)* (129–132)	Liver lipogenesis; Adipogenesis; Insulin resistance; Obesity;, T2D risk	Liver inflammation*;	Liver fibrosis*;

DNL, de novo lypogenesis; T2D, Type 2 diabetes; FA, fatty acids; HC, hepatocarcinoma; KCs, Kupffer cells; HSCs, hepatic stellate cells; MetS, metabolic syndrome; *controversial role, conflicting data.

**Table 4:** Potential effects of the main hepatokines and adipokines on non-alcoholic fatty liver disease pathogenesis (29, 30, 92, 102).

*Organokine*	*Serum level in NAFLD*	*Effect on steatosis*	*Effect on inflammation (NASH)*	*Effect on fibrosis*	*HCC risk*	*Insulin resistance*
***Adiponectin*** (91–93, 133)	Low	Reduces	Reduces	Reduces	No	No
***Leptin*** (79, 93, 97, 134–136)	High	Reduces	Aggravates	Aggravates	Yes	No
***Resistin*** (98, 100, 102, 103, 137)	High/decreased in NASH*	Aggravates	Aggravates*	Aggravates	Yes	Yes
***Ghrelin*** (39, 92, 108–110, 138 *–* 139, 140)	Low	Aggravates	Reduces	Reduces	*	Unclear
***FGF21*** (112 *, * 114, 115, 141)	High (decreased in severe forms)	Reduces	Reduces	Reduces	No	No
***Fetuin A*** (118–121)	High (very high in NASH)	Aggravates	Aggravates	Reduces*	Yes	Yes
Chemerin* (86, 124, 125, 142, 143)	High (reduced in advanced stages of NAFLD)	Aggravates	Aggravates*	Aggravates	Yes	Yes
Visfatin * (127, 144–152)	High*	Reduces*	Aggravates*	Aggravates*	Yes	Yes
***Selenoprotein P*** (129, 131, 132, 153)	High (low in NASH and HCC)	Aggravates*	Aggravates*	Aggravates*	No	Yes

FGF21, fibroblast growth factor 21; NASH,nonalcoholic steatohepatitis; NAFLD, non-alcoholic fatty liver disease; HCC, hepatocellular carcinoma; *variable data.

## 4 HIV and HBV Role in NAFLD Pathogenesis and in the Breakdown of Hepatokine/Adipokine Axis

HIV/HBVco-infection promotes hepatic injuries during NAFLD through several mechanisms triggered by their persistent replication, enhanced inflammatory response and metabolic interference. The toxicity of antiretroviral therapy (ART) can also contribute to NAFLD. Hence, the prevalence of NAFLD can amount to 50-65% of HIV infected patients, depending cumulative risk exposure to metabolic factors, drug toxicities, age, disease duration or ARV regimen (169, 170). By comparison, the prevalence of NAFLD in HBV-infected patients is estimated at 31.4% (171) while HIV/HBV co-infected patients also display a lower prevalence of only 30% (172). On the other hand HIV/HBV co-infected patients show a higher risk of liver fibrosis of 37-40% (173) and exhibit a more rapid progression to liver cirrhosis and HCC compared with HIV mono-infected patients (173, 174). The pathogenic differences between the two viruses explain their different influence on the progression of NAFLD (174). We present below the pathogenic mechanisms associated with the progression to NAFLD in HIV and HBV infection along with the role of antiretroviral treatment (ART) and the ensuing disruption of the hepatokine/adipokine axis.

### 4.1 HIV-Associated NAFLD Mechanisms

HIV promotes NAFLD through multiple mechanisms, as follows: a) the generation of an inflammatory response within the liver; b) the metabolic changes induced in the adipose tissue; c) the disruption of the hepatokine/adipokine axis which maintains the immune and metabolic balance. These mechanisms will be presented below.

#### 4.1.1 HIV- Associated Liver Inflammation

HIV promotes liver inflammation through direct mechanisms or indirectly, following the breakdown of the intestinal immune response during HIV enteropathy.

##### 4.1.1.1 HIV and liver inflammation

**Figure 2:**
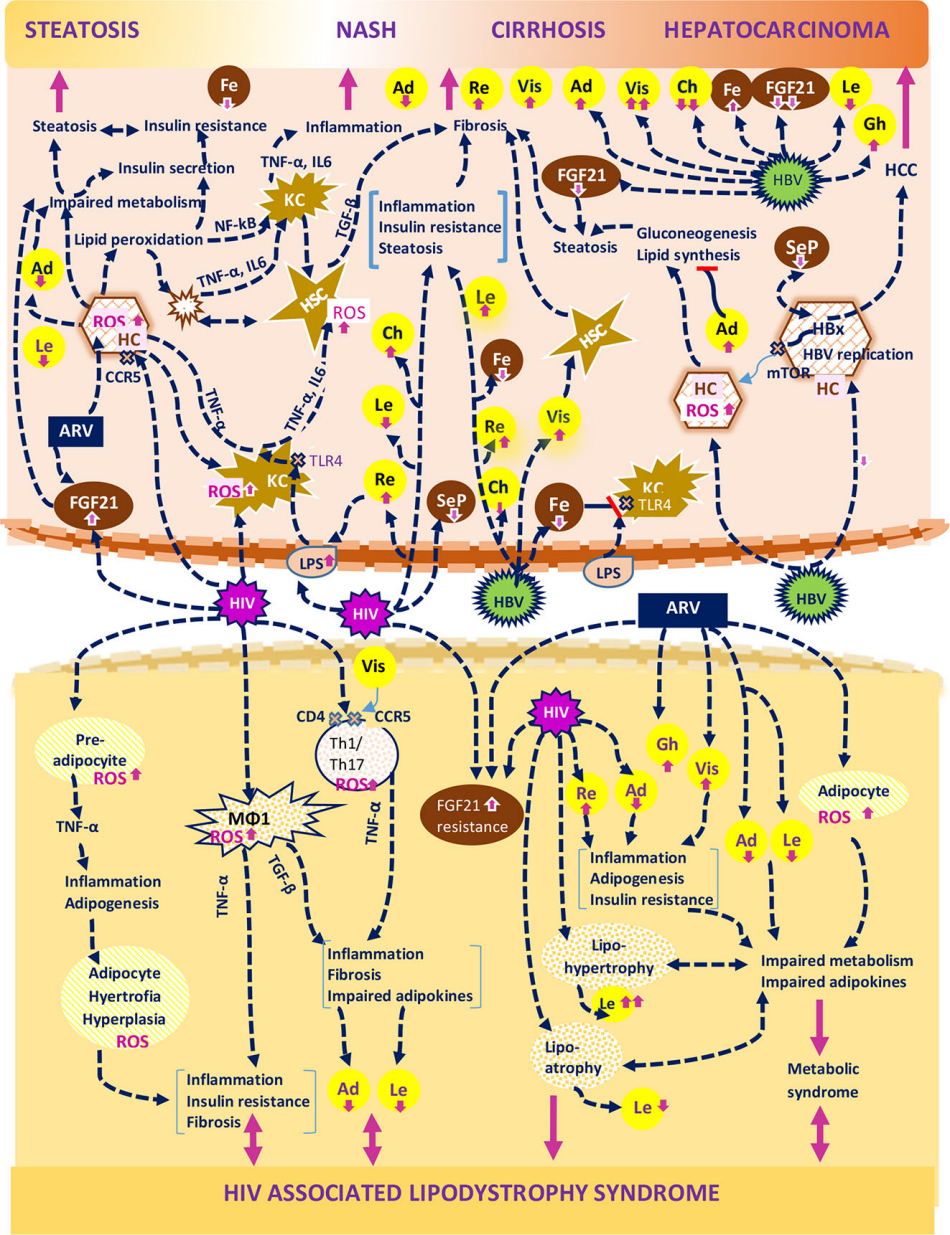
A brief representation of metabolic and inflammatory mechanisms generated by HIV and HBV infections and their interference with the hepatokine/adipokine axis during the progression of non-alcoholic fatty liver disease. The figure shows: a. The effects of HIV on parenchymal liver cells: activation of CCR5 receptors of hepatocytes; the release of reactive oxygen species (ROS) and their subsequent effect on metabolic alterations inducing hepatic steatosis and non-alcoholic steatohepatitis (NASH). ROS excess also promotes lipid peroxidation and hepatocyte necrosis, which in turn aggravate liver inflammation and fibrosis either directly, through proinflammatory cytokines (TNF-α, IL6) and profibrogenic cytokines (TGF-β) or indirectly, through the ensuing proinflammatory and profibrotic response. b. The effects of HIV on non-parenchymal liver cells: both Kuppfer cells (KCs) and hepatic stellate cells (HSCs) can be regulated by HIV directly and indirectly *via* endotoxins (LPS), leading to the progression of the inflammatory response and fibrosis. c. The effects of HIV on adipose tissue cells: HIV ensures the transformation of these cells in cells with pro-inflammatory properties (adipocyte and preadipocyte cells, Th1/Th17-CD4ly lymphocytes and macrophages-MΦ1); HIV also promotes a disproportionate release of hepatokines and adipokines which in turn lead to liver inflammation, adipogenesis, insulin resistance and ultimately to HALS. d. The actions of HBV on hepatocytes: the induction of ROS with metabolic consequences; the changes in the concentrations belonging to hepatokines that promote fibrogenesis and hepatocellular carcinoma (HCC); the decreasing concentration of the protective hepatokine FGF21; the activation of mTOR, a metabolic receptor, stimulated by HBV protein x (HBx) e. The effect of antiretrovirals (ARVs) on the adipose tissue: ARVs favor the release of adipokines with lipogenetic role (e.g. resistin) and the reduction of anti-adipogenic adipokines (e.g. adiponectin, and leptin) f. The impact of HIV and ARVs on the occurrence of a specific metabolic syndrome, namely HIV/ARV associated lypodistrophy syndrome (HALS). HALS arises as a result of HIV-associated inflammatory changes and ARV-related impact on adipogenesis and is mediated by multiple mechanisms (adipocytes hypertrophy or atrophy, the evolution of the metabolic syndrome and the imbalance of hepatokine adipokine axis). Organokines synthesized predominantly in the liver are presented in brown and those synthesized predominantly in the adipose tissue are presented in yellow. The aggravating actions for the liver and adipose tissue are shown in pink. HC, hepatic cells; KC, Kupfer cells; HSC, hepatic stellate cells; ARV, antiretrovirals; Mϕ, macrophage; ROS, reactive oxygen species; HBx, hepatitis B virus X protein; Vis, visfatin; Re, resistin; Fe, fetuin; Le, leptin; Ch, chemerin; Ad, adiponectin; Gh, ghrelin; SeP, selenoprotein; Re, resistin; Th, T helper lymphocyte; LPS, endotoxin; 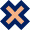 receptor; 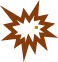 apoptotic cells; TLR4, Toll-like receptor 4.

HIV was detected in the mitochondria of non-parenchymal cells (175), while the infection of hepatocytes was observed only experimentally (176). HIV replication in HSCs and KCs induces mitochondrial toxicity accompanied by inflammation and fibrosis (177). At the same time, HIV releases numerous profibrogenic, proinflammatory and proapoptotic signals in the liver parenchyma (178) which contribute to ROS generation (179), metabolic activation of CD4+T cells and immune stimulation (180). Consequently, HIV induces the cellular death by apoptosis or pyroptosis along with hepatocytes necrosis (181). Additionally, the activation of hepatic T and B lymphocytes during HIV infection and their ensuing apoptosis aggravates the inflammatory response and accelerates the fibrogenesis induced by HSCs (182) (**Figure 2**).

##### 4.1.1.2 HIV enteropathy and inflammatory consequences

In the early stages of HIV infection the viral invasion of the gastrointestinal system weakens the intestinal mucosa through the depletion of CD4+T lymphocytes, thereby promoting liver injury through the microbial translocation of intestinal germs or immunogenic molecules such as LPS, a proinflammatory Gram negative endotoxin. The latter activate KCs through TLR4/CD14 signalling and induce proinflammatory cytokines (TNF-α, IL-1β and IL-6) and profibrogenic mediators (TGF-β) (183). These mechanisms further contribute to neutrophil recruitment and HSCs activation, aggravating liver fibrosis (184). Notably, the inflammatory enteropathy persists and worsens during HIV infection despite ART.

#### 4.1.2 Metabolic Changes Induced by HIV and ART

##### 4.1.2.1 HIV metabolic alterations

HIV promotes NAFLD mostly through its impact on the adipose tissue, an HIV reservoir and a promoter of metabolic alterations in this infection. HIV infects the resident immune cells of the adipose tissue and induces an extensive inflammatory response which leads to the activation of macrophages and pre-adipocytes with macrophage-like properties (185). The release of proinflammatory and profibrotic cytokines change over time the morphology and distribution of the adipose tissue and contributes to the development of HALS, a specific form of lipodystrophy. HALS is characterized by central fat gain along with the loss of adipose tissue in the periphery and weight loss. The metabolic disturbance leads to MetS and to a high cardiovascular risk (186) but is initially independent of ART (187). Later, HALS becomes a complication of ART.

##### 4.1.2.2 ART-metabolic alterations

ART has been a major step towards improving the lifespan of HIV patients. It is undeniable that ART has saved millions of patients who have been given a chance at a normal life. ARV drug classes target various steps of the HIV replication cycle, namely preventing HIV cell entry (Entry inhibitors-EIs), blocking HIV reverse transcriptase (Nucleoside Reverse Transcriptase Inhibitors-NRTIs and Non-Nucleoside Reverse Transcriptase Inhibitors-NNRTIs), impeding protein synthesis (Protease Inhibitors-PIs) and inhibiting the integration of HIV DNA into host DNA (Integrase Strand Transfer Inhibitors-INSTIs). Unfortunately, ART is not completely effective against proviral HIV DNA (188–190) so that it does not provide the eradication of HIV and consequently ART lifelong administration is required. The sustained administration of ART is accompanied by multiple side effects including metabolic changes and HALS (191). The risk of HALS is associated with the duration and type of ART regimen and persists even after ART interruption (192).The exposure to NRTIs is particularly associated with peripheral lipoatrophy while PIs are frequently correlated with visceral lipohypertrophy (193, 194).

The metabolic changes occurring during ART are commonly associated with the cellular toxicity. Hence most ARVs have been shown to induce cumulative cellular toxicity irrespective of the ARV class and independent of HIV stimulation (195, 196). Of these, the most studied and the most aggressive type of cellular toxicity is represented by the mitochondrial toxicity (197) induced by NRTIs, NNRTIs, PIs and by some INSTI representatives (198, 199). This may occur shortly after ART starting and may be perpetuated depending on various risk factors (197, 199–201).

ART induces mitochondrial dysfunction by disrupting the oxidative phosphorylation and ATP synthesis as well as through the excessive release of ROS (196). The OxS generated by ART induces apoptosis, inflammation and fibrosis of the adipose tissue and of liver parenchymal cells. Both mitochondrial dysfunction and the metabolic changes play a decisive role in the development of HALS (194, 202) and favor liver steatosis and NAFLD development (194) as can be seen in the **Figure 2**. The degree of liver toxicity becomes apparent after 6-8 months of NRTI treatment (203) and differs depending on the type of NRTI and its persistence within the cell (200, 201). MetS also develops as a consequence of ART-mitochondrial toxicity (202, 204) and of visceral adipose tissue imbalance induced by hepatokines/adipokines disequilibrium (205). In turn, MetS favors lypodistrophy, increases the cardiovascular risk and contributes to liver steatosis (186, 191, 206) regardless of virological parameters (169).

*In conclusion*, the metabolic abnormalities arising during HIV and ART promote HALS, MetS and liver steatosis (205) and further lead to the development of NAFLD (207). It is estimated that each year of NRTI treatment may add an 11% risk to NAFLD (206). However the progression of NAFLD to liver fibrosis is a rare and lengthy process (208). The metabolic and inflammatory mechanisms generated by HIV and ART are depicted in the **Figure 2**.

#### 4.1.3 Hepatokine/Adipokine Axis Breakdown in HIV-Specific NAFLD

In its attempt to restore the metabolic balance, and to reduce the HIV-lipodystrophy and IR, the liver regulates the metabolism of the adipose tissue by activating the hepatokines and adipokines circuit. Unfortunately, there is limited data regarding the hepatokines/adipokines roles in HIV patients. Currently, the most documented organokines involved in body fat distribution are leptin and adiponectin, two adipokines mainly secreted by adipocytes. Available data on adiponectin indicate that ART and in particular the treatment with IPs (ritonavir), lowers the levels of adiponectin and leptin especially at the beginning of therapy and favors the progression of lipodystrophy, steatosis and IR (195, 209, 210). In turn, the development of lipodystrophy lowers the concentration of these two adipokines independent of the ARV agent (211–214). Hence, an effective treatment against lipodystrophy could normalize the level of these two adipokines and could additionally lower the risk of steatosis. Recent studies indicate that the reduction of the serum adiponectin and leptin levels could be a consequence of SIRT1-depleted adipocytes found during lipodistrophy (215, 216). SIRT1, a member of the enzyme family of sirtuins, modulates the cellular metabolism, as well as HIV transcription (217). SIRT1 downregulates DNL and gluconeogenesis improving the lipid and carbohydrate metabolism, and protecting the liver from steatosis (218). The inhibition of SIRT1 along with the decrease of adiponectin and leptin expression during HIV infection as well as during PIs treatment could represent one of the steps required for the development of HIV-associated NAFLD (219). On the other hand, experimental data suggest that HIV could promote the release of adiponectin (211). Therefore HIV suppression during ART may lower the levels of adiponectin which would partly explain the metabolic changes associated within HALS (211). In this regard, HIV-infected patients starting ART may still develop MetS and NAFLD and may progress to HCC eventually (91, 220). Leptin is a liporegulatory adipokine which can display a protective role on pancreatic beta cells and liver steatosis but also increase the risk of inflammation in non-HIV infected patients (221, 222). As a result hipoleptinemia is linked to a lower risk of inflammation, fibrogenesis and carcinogenesis (96, 134, 223). On the other hand, in HIV-infected patients, the leptin secretion is closely related to the body fat mass, so that leptin deficiency prevails in cases with lipoatrophy, while hyperleptinemia accompanies patients with lipohypertrophy (212, 224). The treatment of obesity with rosiglitazone, an inhibitor of the leptin release could potentially reduce the hyperleptinemia in HIV infected patients improving metabolic parametric and the cardiovascular risk (225). The administration of leptin in hipoleptinemia could also contribute towards a metabolic balance but this type of therapy is not yet available in HIV patients. Human recombinant leptin (metrelin) is the only organokine that has been approved in the treatment of non-HIV associated lipodystrophy. Certain studies have suggested that two months of metreleptin treatment can also improve lipodistrophy and the metabolic syndrome including in patients undergoing ART (226, 227). However, in HIV patients the proinflammatory and profibrotic effect of leptin remains unknown. Therefore leptin treatment seems to be hazardous especially that serum leptin levels may be associated with leptin resistance, severe forms of NAFLD and HCC risk (135). Recombinant leptin has also been studied in NASH non-HIV patients as part of a clinical trial ( ClinicalTrials.gov Identifier: NCT00596934), but it has not been studied in HIV/NAFLD patients. Nevertheless, there are still insufficient data to recommend this therapy in NAFLD patients.

Adiponectin has also been studied in both cell cultures and mice. Hence, the supplementation with adiponectin alleviates the metabolic syndrome, IR and steatosis and could antagonize the oncogenic effects of leptin against the liver (220, 228, 229). Additionally, adiponectin agonists have been used as an experimental antifibrotic therapy in liver diseases (230). It is considered that hypolipidemic therapies such as statins and antidiabetic medications thiazolidinediones or metformin could upregulate the level of adiponectin and induce a hepatoprotective effect without side effects (231, 232). However, there are conflicting data on the use of these drugs in HIV (212, 233–235) and the development of resistance to leptin and adiponectin limits their activity even in the presence of high serum levels (212, 236).

Data on other hepatokines and adipokines in HIV infected patients is summarized below.

SeP, a hepatokine with antioxidant properties during HIV activation and ROS release (237, 238) has been shown to display low levels in HIV patients. On the other hand, non-HIV patients with NAFLD display high serum concentrations of SeP, which are associated with the metabolic disturbances and NAFLD progression (129). FGF21, a hepatokine with a key metabolic role in glucose and lipid homeostasis is also expressed in HIV patients. The detection of a high FGF21 serum level in these patients was associated with MetS, lipodistrophy, and severe steatosis (239–241). Given the strong correlations between FGF21 and metabolic parameters, FGF21 has been suggested as a possible prognostic marker to monitor HALS and its therapy as well as the ART-associated cardiovascular risk (242). HIV infected patients also show high serum levels of resistin, an adipokine correlated with IR, lipoatrophy and NAFLD (243). In addition, it has been observed that genetic variants of resistin could play a role in the progression of HALS (244). HIV lipodistrophy also appears to be promoted by another regulator of adipogenesis, namely chemerin, an immunomodulatory adipokine, also with proinflamatory activity in NAFLD (88). Experimental data suggested that CMKLR1/ChemR23, a specific chemerin receptor could be used by HIV as a minor coreceptor but this remains to be confirmed (245). Visfatin, an adipokine released by various cells within the adipose and liver tissue interacts with a C-C chemokine receptor type 5 (CCR5), which is an HIV coreceptor but the result of this interaction is presently unknown (246, 247). Another organokine, namely ghrelin, has shown discordant results in HIV lipodystrophic patients (248, 249) The serum level of ghrelin has been concordant with the serum level of TGs on a small group of patients. Nevertheless, the administration of ghrelin was associated with the attenuation of liver inflammation in mouse models (250).

Overall, data on the direct impact of hepatokines and adipokines during HIV infection on ART is scarce and their role in the progression towards NAFLD remains unclear. The disruption of the hepatokine/adipokine axis during HIV infection and the ensuing implications for NAFLD progression are shown in the **Figure 2** and **Table 5**.

**Table 5:** The main hepatokines and adipokines discussed in the article and their implications in HIV and HBV infection.

*Organokine*	*NAFLD (serum levels)*	*HIV infection (serum levels)*	*HBV infection (serum levels)*
***Adiponectin***	Low	Low serum level is associated with lypodistrophy, insulin resistance and dyslipidemia (248). ART reduces the level of adiponectin and its effect is more significant in patients with hypertriglyceridemia (247, 251). Adiponectin supplementation may improve ART-induced metabolic changes (210, 212)	*High serum* adiponectin levels in cirrhosis, correlated with liver dysfunction. High serum levels in HBV obese patients with positive HBV viral load *Liver* adiponectin level is high in areas of HBV necro-inflammation, and low in fibrosis (252, 253)
***Leptin***	High	Low serum level is associated with liver steatosis, lipoatrophy and insulin resistance (248, 254); high level was observed in lipohypertrophic patients (leptin resistance)?. Treatment with leptin improves some metabolic components of lypodistrophy induced by ART (255)	High level in chronic HBV (256) Low level in HCC and cirrhosis with malnutrition; negative correlation with TNF-α (256, 257)
***Resistin***	High	High level. Certain genetic polymorphisms of resistance are associated with HALS (244); HIV patients with lipoatrophy and insulin resistance may respond to treatment with PPAR-γ agonists (243)	High level in chronic HBV (index of disease severity) (258)
***Ghrelin***	Unknown	Low level in HIV patients * High level in ART-related hypertriglyceridemia (251)	High level in HCC and cirrhosis with malnutrition; positive correlation with TNF-α (259)
***FGF21***	High	Very high level (resistance or compensatory effect)? associated with HALS, insulin resistance, severe steatosis. Very high level post ART (marker of lipodystrophy) (242)	Low level in chronic HBV and cirrhosis High level in HCC (260)
***Fetuin A***	High	No studies	Low level in HBV Very high level in HCC (261) Predictor of NASH
***Chemerin***	High	A possible minor co-receptor in HIV (245)	Low level in chronic HBV Very low level in HCC/HBV (262) Therapeutic potential of chemerin in HCC (262)
***Visfatin***	High	High level in ART (247)	High serum levels in cirrhosis Very high levels in HCC (263) Marker of necroinflammation
***Selenoprotein P***	High	Low level (238); predictor of HIV survival (264–266)	Low level Serum level is associated with HBx overexpression (267)

HBV, hepatitis B virus; HCC, hepatocarcinoma; HALS, HIV/ART–associated lipodystrophy syndrome; NAFLD, non-alcoholic fatty liver disease; NASH, stetatohepatitis; ART, antiretroviral therapy; *divergent studies.

### 4.2 HBV-Associated NAFLD

HBV is a hepatotropic DNA virus and a major cause of cirrhosis and HCC in NAFLD patients. It is transmitted through the same pathways as HIV and infects about 10% of these patients (268). The HBV co-infection with an immunosuppressive virus such as HIV further reduces the ability of the immune response to recognize and eliminate HBV-infected cells. Thus HIV infection promotes HBV replication along with the synthesis of proinflammatory and profibrotic cytokines. In turn, HBV facilitates the ongoing HIV viral replication and impacts the recovery of CD4+T cells during ART (268–274). Furthermore, the use of NRTIs as a treatment in HIV/HBV patients could aggravate the mitochondrial dysfunction, inflammatory response (275) and favor additional metabolic complications. As a result, HIV-HBV co-infected patients display a more rapid progression towards liver cirrhosis and HCC (268, 272). Nevertheless, HBV appears to attenuate various metabolic effects induced by HIV and ART, so that the prevalence of NAFLD in HIV/HBV patients is lower than in HIV or HBV monoinfected patients (169, 170, 172). The mechanisms by which HBV influences the pathogenesis of NAFLD are depicted below.

#### 4.2.1 HBV-Induced Hepatic Inflammatory Response

HBV is not a cytopathic virus; it generates 3 types of antigens that interfere with the immune response, preventing the recognition and elimination of virally infected hepatocytes. Of these, HBsAg is a modulator of the innate immune response which contributes to the suppression of inflammatory cytokines, the aggravation of liver fibrosis and the risk of malignant transformation (276, 277). HBcAg induces the secretion of IL18, a potent proinflammatory cytokine engaged in hepatocytes pyroptosis, one of the profibrotic mechanisms in HBV infection (278). HBeAg, the third viral antigen inhibits NF-κB pathway and ROS production in KCs and consequently suppresses NLRP3 inflammasomes and the inflammatory response (279). The inhibition of the inflammatory response allows HBV to escape the immune response and to induce a persistent infection along with HBV DNA insertion into the host genome, which further adds to the risk of oncogenesis.

#### 4.2.2 HBV-Induced Metabolic Dysfunction

HBV has been viewed as a ‘‘metabolic virus’’ with multiple metabolic interferences (280). Still the correlation between HBV and the metabolic changes in the pathogenesis of NAFLD remains unclear. The metabolic dysfunctions are initiated as the same time with viral replication due to the activation of some transcription factors and nuclear receptors (281). These regulatory proteins once activated in the early stages of HBV transcription increase the expression of key enzymes involved in the control of metabolic pathways such as lipolysis, gluconeogenesis and cholesterol synthesis (281, 282). The activation of metabolic-related transcription factors is related to the overexpression of hepatitis B virus X protein (HBx), a regulatory protein involved in FA metabolism, steatosis and adipogenesis (282, 283). In addition to its metabolic contribution Hbx is a transcriptional activator mediating both NF-kB/TNF-α inflammatory signalling and cellular apoptosis, further contributing to DNA damage and liver carcinogenesis. Hence, HBx targets multiple mechanisms involved in the progress of NAFLD (284–286). However despite experimental evidence, the correlations between HBV and hepatic steatosis remain inconstant contradictory or even negative (284, 287, 288).

Studies on HBV infected patients have indicated that MetS appears to evolve independently of the HBV viral infection (289, 290). However, the presence of the metabolic changes once triggered (obesity, dyslipidaemia, diabetes) and the persistence of these changes supports the ongoing HBV viral replication. In turn, viral replication promotes hepatic steatosis and fibrosis, both of which contribute to the continuous progression of the metabolic dysfunction (280, 291–296). The duration of the MetS and of HBV infection may also influence the onset of steatosis (297).

In conclusion, HBV mainly promotes hepatic steatosis as well as liver fibrosis and carcinogenesis, yet it is less associated with liver inflammatory changes (**Figure 2**). By comparison, HIV induces early inflammation and irreversible metabolic changes further boosted by ART, so that during HIV and HBV coinfection both viruses play additive roles in the evolution of NAFLD (10, 298). It should be noted that both viruses also change the cellular metabolism through the conversion of glucose to lactate and further perpetuates the lactate synthesis in the presence of oxygen similar to the cancer cells (Warburg effect) (299). In this respect, HBV has been significantly linked to carcinogenesis, while HIV has been shown to strongly modulate the immune response and MetS.

#### 4.2.3 Hepatokine/Adipokine Axis Breakdown in HBV Patients With NAFLD

There are few data on the hepatokine/adipokine axis in HBV infected patients. Studies on HBV infected patients have shown links between elevated levels of some hepatokines and insulin inhibition (e.g fetuin), between hepatokines, gluconeogenesis and the inflammatory response (fetuin A, leptin, resisitin, visfatin), between hepatokines and steatosis (leptin) as well as between hepatokines and HCC evolution (ghrelin, FGF21, fetuin A) (**Table 2**).

The hepatokines best documented in HBV infected patients include fetuin A and FGF21.

Fetuin A is a hepatokine predominantly released by the liver and correlated with the development of both MetS and NAFLD. The serum level of fetuin A is significantly higher in NAFLD but decreases in liver failure along with the extension of hepatic necrosis. A low level of fetuin A in HBV infected patients has been proposed as a marker of liver damage, as well as a predictive factor for poor prognosis (300). However, the highest fetuin A serum levels have been recorded in HBV patients with HCC and cirrhosis (261) while fetuin A levels in non-HBV patients has not been associated with fibrotic changes (118). Furthermore experimental data on HBV infected patients has shown that fetuin A attenuates the pro-inflammatory response induced by LPS administration (300). Thus, a low fetuin A secretion which follows hepatocyte necrosis explains the ensuing hepatic inflammatory response and the development of NASH, while fetuin A supplementation could alleviate the inflammatory response (301).

Another hepatokine that reflects the presence of liver damage is FGF21 secreted mainly in the liver but active especially in the adipose tissue. FGF21 is considered a stress hepatokine (302) with antioxidant and anti-inflammatory potential (303, 304). FGF21 secretion is reduced in chronic HBV infection, particularly in cirrhosis and the administration of FGF21 in HBV infected patients could improve liver inflammation and fibrosis (34, 304, 305). However FGF21 serum levels may increase in liver injuries and a very high level of FGF21 has been associated with HCC risk possibly as a protective response against the carcinogenesis process (306). Moreover, a high FGF21 serum level could be considered a marker of severity in patients with chronic HBV (260). A moderately elevated level has also been observed in NAFLD in non-HBV patients (307) in which case the FGF21 concentration has been correlated with metabolic improvement especially in the insulin response (308). Overall, the increased secretion of FGF21 arises as a compensatory response to OxS, and therefore a high level has a limited efficiency in patients with chronic HBV.

Certain adipokines with a fibrogenetic and neoplastic potential display high serum levels in HBV infected patients but their levels decrease during treatment, along with HBV viremia (30). Such is the case of leptin, a proinflammatory adipokine produced in HSCs and correlated with NAFLD fibrosis, which decreases during lamivudine HBV treatment (309).

Adiponectin is a metabolic, anti-inflammatory and antifibrotic adipokine. Experimental data indicate an improvement in steatosis, inflammation and liver fibrosis after adiponectin administration (228). Patients with chronic HBV infection display a high level of adiponectin and this level decreases during interferon therapy along with HBV viral load (253, 310). In these patients the level of adiponectin correlates with various stages of liver injury from liver inflammation and fibrosis to HCC (67, 311). Current reports indicate that adiponectin may promote HBV polymerase activity and HBV DNA replication, while in turn HBV replication induces the expression of adiponectin (312). This observation could partly explain the correlation above, as well as the hypothesis that adiponectin may play a role in the progression of HBV liver injury (252). The level of adiponectin differs in patients with HBV compared with non-HBV/NAFLD patients where, for example, the level of adiponectin decreases with the development of MetS and NASH yet may increase in patients with cirrhosis regardless of metabolic factors (133, 252, 313). In other studies, however, liver histology indicates a correlation between adiponectin and steatosis but not between adiponectin and viral factors (311).

Regarding other hepatokines and adipokines, chemerin is a multifunctional hepatokine with antioncogenic properties against HCC metastases, yet with reduced activity in HBV-HCC tissues (314). The low concentrations of chemerin in HCC-HBV infected patients have been associated with a favorable prognosis, thus suggesting a potential therapeutic role for this hepatokine. Nevertheless, the intratumoral level of chemerin was variable across different studies and experimental data on the matter are contradictory (262, 314). Resistin is another adipokine which regulates the development of obesity and IR and which has been correlated with the severity of liver damage in HBV infection (258, 315).

The imbalance of the hepatokine/adipokine axis in HBV infected patients and its implications in the progression of NAFLD are shown in **Figure 2** and **Table 5**. In most cases the serum levels of adipokines released in HBV infected patients resemble the levels reported in other non-HBV infected patients with NAFLD, as seen in the cases of visfatin, leptin and resisitin. Regarding HIV infected patients, the serum levels of hepatokines or adipokines could be comparable to those detected in HBV as reported for SeP, or could exhibit a marked difference as for certain adipokines (leptin, adiponectin) or hepatokines (FGF21). These differences could be related to one of the following factors, namely the type of tissue damage (the lipodystrophy and adipose tissue damage predominate in HIV while the liver necrosis predominates in HBV), the incidence of liver fibrosis (more common in HBV), the degree of liver inflammation (more pronounced in HIV), the genetic polymorphisms of certain organokines (316) or the level of viral replication (211).

## 5 Conclusion

The liver contains a wide variety of cells through which it plays a complex role in the glycolipid metabolism, drug excretion and immune response. In this context, the metabolic changes, the treatments with hepatic metabolized drugs and the extent of the immune response have a direct impact on the structure and function of the liver parenchyma. One of the consequences of the immunometabolic imbalance is the development of NAFLD which gradually evolves from fatty liver and insulin resistance to inflammation, fibrosis and even to hepatic carcinogenesis. NAFLD in HIV/HBV co-infected patients could have a high severity due to the potential of the two viruses to replicate and to induce an inflammatory response. In addition both HIV and HBV play an important role in disrupting the glycolipid metabolism and liver communication through the imbalance of the hepatokines and adipokines. The two viruses act complementary and play an additive role in the emergence and progression of NAFLD. At the same time, ART aggravates the metabolic context induced by the two viruses, through the toxic effect on mitochondria and the development of lipodistrophy.

The HIV/HBV correlations with the breakdown of the hepatokine/adipokine axis during the progression of NAFLD are complex and largely unknown. Additional studies with comparable characteristics are needed to better formulate the assumptions of this axis and to further validate the potential therapeutic applications of hepatokines and adipokines.

## Author Contributions

SI and DI contributed equally to the acquisition, analysis, and critical revision of the article. The authors had given their consent for the publication and agreed to be responsible for the accuracy and integrity of the article.

## Conflict of Interest

The authors declare that the research was conducted in the absence of any commercial or financial relationships that could be construed as a potential conflict of interest.

## Publisher’s Note

All claims expressed in this article are solely those of the authors and do not necessarily represent those of their affiliated organizations, or those of the publisher, the editors and the reviewers. Any product that may be evaluated in this article, or claim that may be made by its manufacturer, is not guaranteed or endorsed by the publisher.
